# Chemical structure of hollow carbon spheres and polyaniline nanocomposite

**DOI:** 10.1016/j.dib.2018.01.099

**Published:** 2018-02-03

**Authors:** Linghao He, Bingbing Cui, Jiameng Liu, Yingpan Song, Minghua Wang, Donglai Peng, Zhihong Zhang

**Affiliations:** Henan Provincial Key Laboratory of Surface & Interface Science, Zhengzhou University of Light Industry, Zhengzhou 450002, China

## Abstract

In this data article, the chemical data of hollow carbon spheres and polyaniline (HCS@PANI) nanocomposite are presented for the research article entitled “Novel electrochemical biosensor based on core-shell nanostructured composite of hollow carbon spheres and polyaniline for sensitively detecting malathion” (He et al., 2018) [1]. The data includes chemical structure and components obtained by Raman spectra, X-ray photoelectron spectroscopy (XPS), and nitrogen adsorption and desorption isotherms.

**Specifications Table**

**Table:** 

Subject area	*Chemistry*
More specific subject area	*Biosensor Material*
Type of data	*Figures*
How data was acquired	Raman spectra were taken with Renishaw inVia-Raman Spectroscopy, equipped with a holographic grating of 1800 lines mm^−^^1^ and a He-Ne laser (632.8 nm) as an excitation source.
	X-ray photoelectron spectroscopy (XPS) analysis was obtained from an AXIS HIS 165 spectrometer (Kratos Analytical, Manchester, UK) with a monochromatized Al KR x-ray source (1486.71 eV photons).
	The N_2_ adsorption-desorption isotherms were conducted using a Micromeritics ASAP 2010 instrument with a liquid nitrogen at the temperature of 77 K. The specific surface area was calculated by the Brunauer-Emmett-Teller (BET) method.
Data format	*Analyzed*
Experimental factors	*The samples were ground evenly before measurements*
Experimental features	*The chemical structure and elemental components were examined.*
Data source location	*Zhengzhou University of Light Industry, Zhengzhou 450002, China.*
Data accessibility	*Data are presented in this article*

**Value of the data**

● The data presented in this article shows detailed chemical structure of HCS@PANI nanocomposite.
● This data allows other researchers to compare the preparation of HCS@PANI nanocomposite.
● For fabricating nanocomposites with other functional materials provides a suitable way for biosensors application.

## 1 Data

**Fig. 1:**
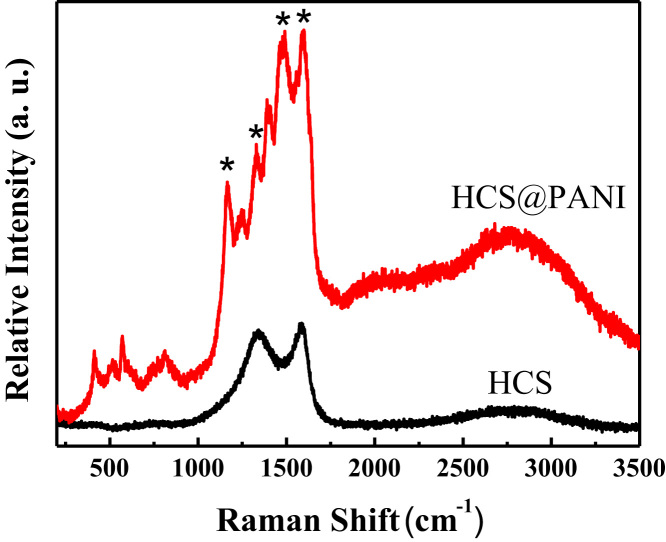
Raman spectra of HCS and HCS@PANI nanocomposite.

The chemical structure, surface morphologies, and electrochemical performances of HCS@PANI nanocomposite were discussed the our previous work [1]. Raman spectra of the as-prepared HCS and HCS@PANI nanocomposite are shown in Fig. 1. For the pure HCS sample, the G band at 1591 cm^−1^ and D band at 1338 cm^−1^ are observed, which correspond to graphitic carbon and disordered carbon, respectively [2]. In case of HCS@PANI nanocomposite, four major peaks corresponding to characteristic of the presence of PANI can be observed (marked by asterisks in Fig. 1). These are i) C–H bending of the quinoid ring at 1166 cm^−^^1^, ii) C–N^•+^ stretching vibration of cation radical species at 1330 cm^−^^1^, iii) C=N stretching at 1480 cm^−1^, and iv) C–C stretching of the benzene ring at 1596 cm^−1^ [3], [4].

**Fig. 2:**
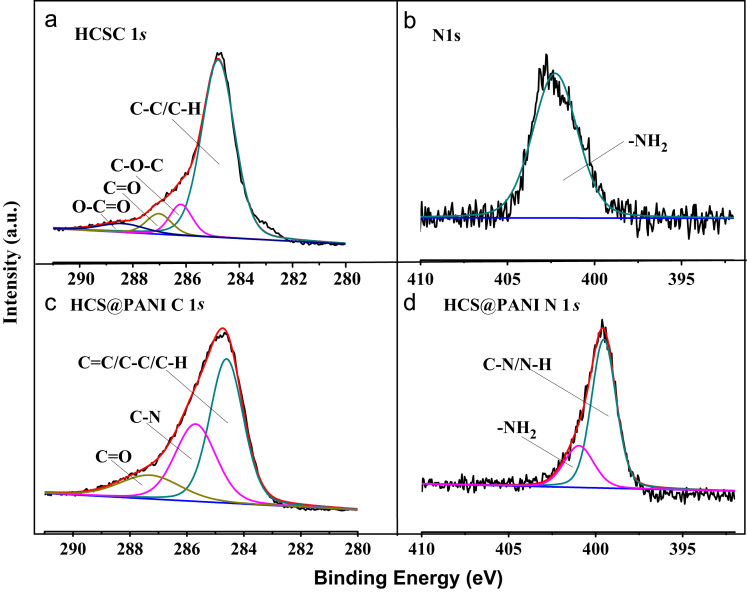
(a, b) C 1*s* and N 1*s* core-level XPS spectra of HCS and (c, d) C 1*s* and N 1*s* core-level XPS spectra of HCS@PANI nanocomposite.

The C 1*s* and N 1*s* core-level XPS spectra of HCS and HCS@PANI were summarized in Fig. 2. The C 1*s* core-level XPS spectrum of HCS (Fig. 2a) is composed of four components, indicating different chemical environments present in the HCS. The peaks at ~ 284.8, ~ 286.2, ~ 287.0, and ~ 288.6 eV are assigned to C–C/C–H, C–O–C, C=O, and O–C=O groups, respectively. In case of HCS@PANI nanocomposite, The peak at ~ 285.9 eV is attributed to C–N group (Fig. 2c). The N 1*s* core-level XPS spectrum was observed in HCS (Fig. 2b). The peak at ~ 401.6 eV is assigned to –NH_2_ groups in the sample, which comes from ammonium hydroxide in the preparation process of HCS. Similar to the N 1*s* core-level XPS spectra (Fig. 2d), two peaks are fitted at ~ 399.0 and ~ 400.8 eV, which are corresponding to the functional groups of C–N/N–H and –NH_2_ respectively.

**Fig. 3:**
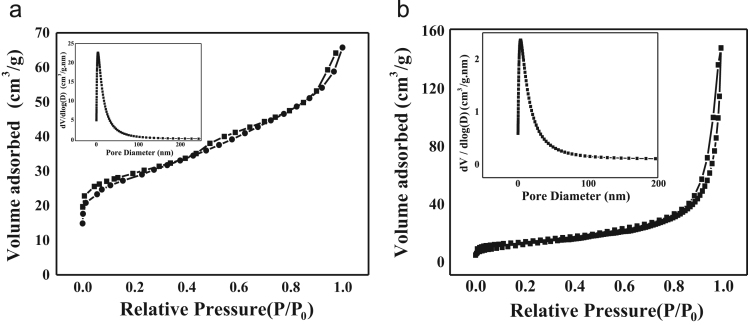
N_2_ adsorption-desorption isotherms of (a) HCS and (b) HCS@PANI, insets: the corresponding pore size distribution.

N_2_ adsorption-desorption isotherms was carried out on the HCS and HCS@PANI nanocomposite (Fig. 3). The isotherm profile exhibits feature of typical type IV with a big hysteresis loop, which is characteristic of mesopores. The BET specific surface area for HCS is evaluated around 75.7 m^2^ g^−^^1^ and an average pore size of 4.6 nm obtained from BET method, while the BET specific surface area for HCS@PANI is around 42.67 m^2^ g^−1^.

## 2 Experimental design, materials, and methods

### 2.1 Synthesis of HCS and HCS@PANI nanocomposite

HCS was prepared by following procedure. In a typical experiment, 30 mL ammonium hydroxide was added drop-by-drop to the mixture of Milli-Q water (10 mL) and 70 mL anhydrous ethanol. After stirring for 30 min, tetraethyl orthosilicate, resorcinol and methyl aldehyde were added into the solution followed by vigorous stirring for 24 h. Afterward, the mixture was transferred into a Teflon-lined stainless-steel autoclave, which was heated to 100 °C and maintained at this temperature for 10 h. Then, the resulting solid was put into the tube furnace and maintained at 750 °C for 1 h. Finally, the obtained powder was transferred to the hydrofluoric acid and immersed for 10 h to etch the formed SiO_2_ nanospheres.

After adding 20 µL of aniline into 20 mL of hydrochloric acid and stirring continuously, HCS (30 mg) was added into the mixed solution and stirred for 30 min. Subsequently, 10 mL of ammonium persulphate (0.1 M) was immersed in the above solution and kept for stirring for 12 h. At last, the atrovirens powder was collected and washed for five times with Milli-Q water. As such, the HCS@PANI nanocomposite was obtained.

### 2.2 Characterizations

X-ray diffraction patterns were recorded on a D8 Advance X-ray diffractometer with CuKα radiation (XRD, Bruker, Germany). X-ray photoelectron spectroscopy (XPS) analysis was obtained from an AXIS HIS 165 spectrometer (Kratos Analytical, Manchester, UK) with a monochromatized Al KR x-ray source (1486.71 eV photons).
