# Supplementary material for: Chemical structure of hollow carbon spheres and polyaniline nanocomposite
Source: Data Brief. 2018 Feb 3;17:796–800. doi: 10.1016/j.dib.2018.01.099 (PMC5988495; doi:10.1016/j.dib.2018.01.099)
Supplement: Supplementary file 1 [file mmc1.docx]

**Conflict of interest**

The author declares no Conflict of Interest.
